# Frequency and Temperature Dependence of Fabrication Parameters in Polymer Dispersed Liquid Crystal Devices

**DOI:** 10.3390/ma7053512

**Published:** 2014-05-02

**Authors:** Juan C. Torres, Ricardo Vergaz, David Barrios, José Manuel Sánchez-Pena, Ana Viñuales, Hans Jürgen Grande, Germán Cabañero

**Affiliations:** 1 Grupo de Displays y Aplicaciones Fotónicas, Departamento de Tecnología Electrónica, Universidad Carlos III de Madrid, C/Butarque, 15, Leganés E28911, Madrid, Spain; E-Mails: rvergaz@ing.uc3m.es (R.V.); dbarrios@ing.uc3m.es (D.B.); jmspena@ing.uc3m.es (J.M.S.-P.); 2 Centre for Electrochemical Technologies (CIDETEC), Departamento de Nuevos Materiales, Parque Tecnológico de San Sebastián - Paseo Miramón, 196, San Sebastián E20009, Spain; E-Mails: avinuales@cidetec.es (A.V.); hgrande@cidetec.es (H.J.G.); gcabanero@cidetec.es (G.C.)

**Keywords:** polymer dispersed liquid crystals, equivalent circuit, constant phase element, temperature dependence, impedance analysis

## Abstract

A series of polymer dispersed liquid crystal devices using glass substrates have been fabricated and investigated focusing on their electrical properties. The devices have been studied in terms of impedance as a function of frequency. An electric equivalent circuit has been proposed, including the influence of the temperature on the elements into it. In addition, a relevant effect of temperature on electrical measurements has been observed.

## 1. Introduction

Polymer dispersed liquid crystals (PDLC) devices consist of a thin film of a polymer matrix containing micro-sized droplets of a liquid crystal. In order to fabricate PDLC devices, the mixture is usually laid between two transparent substrates coated with a conductive layer, usually of indium tin oxide (ITO) [1]. These PDLC devices can be used in eletro-optic applications such as: smart windows [2], variable optical attenuators (VOAs) [3] and projection displays [4].

In order to use PDLC devices as an optical switcher, the refractive index of the isotropic polymer and the ordinary refractive index of the liquid crystal should be similar [5]. In the absence of an electric field, the directors of the microdroplets are randomly distributed and strongly scatter the light so the device appears opaque. When an AC electrical field is applied, the liquid crystal dipoles are re-oriented parallel to the field and the ordinary refractive index of the liquid crystal matches with the polymer refractive index, and the material becomes transparent [6]. The transmission of light through the device, therefore, can be controlled by applying an electrical field. Furthermore, unlike with other electro-optic devices like twisted-nematic LC (TNLC) or surface-stabilized ferroelectric liquid crystal (SSFLC), the transparent state can be highly transmissive because no polarizers are required to achieve the switching effect.

PDLC has a great number of advantages in comparison with the other liquid crystal technologies used in displays, such as: high brightness (because of its high transparency), wide view angle, fast response (in the order of milliseconds), absence of surface treatment and the possibility of intermediate transmission levels electrically controllable. The scattering properties of PDLC devices can be used in “smart” windows that regulate the light intensity inside buildings. The shift with temperature of the electrooptic response curve of the PDLC is well known [6]. When using these devices with intermediate transparency levels rather than in ON/OFF mode, the knowledge of the temperature variation is mandatory in a double way: to optimize the contrast and to estabilize the transparency level [2].

Therefore, in order to ensure a determinate transmission level, it is necessary to change the electrical field applied to the PDLC according to the ambient temperature. The most practical way to mitigate the temperature influence in an effective way is to control the electrical field, as the optical properties are mainly related to it.

On the other hand, an electrical equivalent circuit (EEC) associated to electrical properties (frequency, impedance and voltage) that also varies with temperature is of great practical interest, because it can be used to develop new driving circuits and signals in order to reduce power consumption and increase optical contrast.

In this work, the electrical properties of a series of PDLCs have been analyzed. This behavior can be represented through an adequate combination of passive elements which responses are associated to physical parameters. An electrical equivalent circuit describing such behavior has been proposed. The component values of the circuit have been obtained from complex impedance measurements. Experimental results and simulation have been compared in order to validate the EEC. The aim of the work is to explore the temperature dependence of the equivalent circuit electrical parameters of the device. This knowledge allows, then, a practical way to control the optical performance of the device regardless of the temperature.

## 2. Experimental Setup

The PDLCs devices were manufactured from an homogeneous mixture of 20 wt% of a UV curable matrix (Bisphenol A glycerolate diacrylate from Aldrich) and 80 wt% of an eutectic nematic liquid crystal mixture (E7 from Merck) consisting mostly of 4-pentyl-4’-cyanobiphenyl, with positive dielectric anisotropy. Glass cells with ITO coated inner surfaces with an effective area of 1.3 × 1.3 cm^2^ and a 20.5 μm internal gap, were filled by capillarity with the mixtures at 65 °C. PDLC was formed by polymerization induced phase separation (PIPS) method, exposing the cell to a 365 nm light for 10 min. The UV flux was 350 mW/cm^2^ at a distance of 15 cm (Vilber Lourmat VL4LC, Marne La Vallee, France).

**Figure 1.**
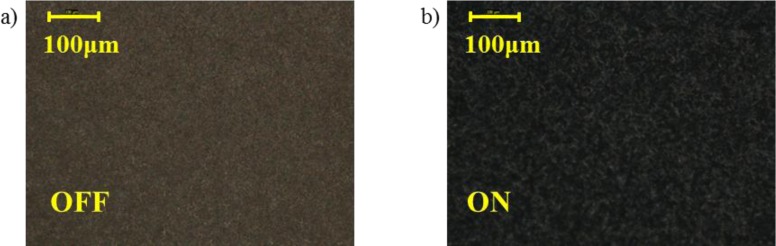
Micro-textures of the droplets morphologies of the polymer dispersed liquid crystals (PDLC) films under cross polarizers at 20× magnification and PDLC devices in the OFF and ON state: (**a**) V_RMS_ = 0 V; (**b**) V_RMS_ = 24 V.

The key to obtain the orientation is the observation of the device by polarization microscopy, observing the pattern of the droplets with the sample located between two crossed polarizers [6]. The morphology of the PDLC composites was studied at 20× magnification using a Leica DM400M microscope (Leica Microsystems, Wetzlar, Germany), a Leica camera DFC420C (Leica Microsystems) and placing the devices between crossed polarizers (Figure 1, the scale bar corresponds to 100 μm). Unfortunately, the droplets are too small to be resolved. The small size can be associated to a fast polymerization and phase separation process because of UV curing at high temperature (65 °C) as it is reported elsewhere [7,8].

Afterwards, the measurement of complex impedance (magnitude and phase) was carried out using an impedance analyzer (SOLARTRON 1260, Solartron Analytical, Farnborough, UK), in a wide frequency range, 0.1 to 10 MHz. PDLC devices have been placed in a programmable hot-stage (Linkham plate LTS-E350, Linkam Corp., Surrey, UK) in order to guarantee a stable temperature during a whole measurement process. The sample temperature range was measured from 25 to 40 °C with a change of 5 °C per measurement.

## 3. Impedance Behavior

**Figure 2.**
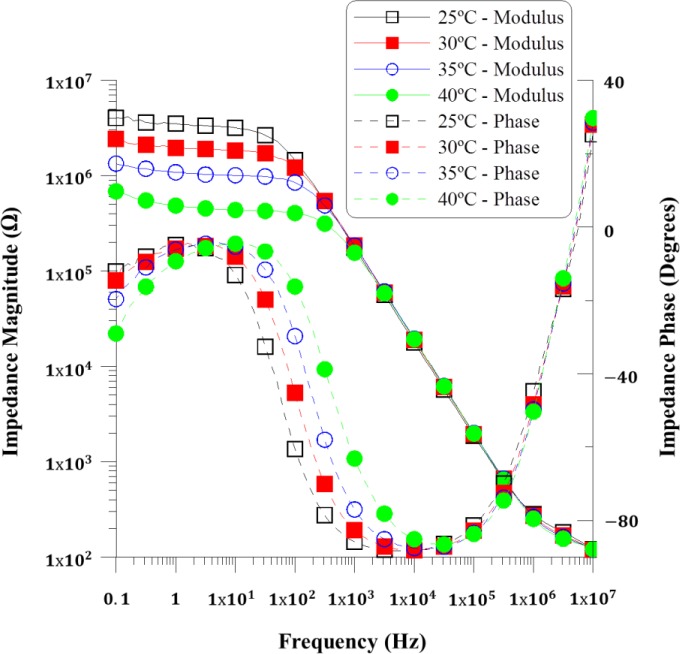
Experimental complex impedance as a function of frequency in the temperature range between 25 °C and 40 °C.

The complex impedance was measured using a sinusoidal voltage signal with 100 mV_RMS_ and frequency sweeping in the range from 10^−1^ to 10^7^ Hz. The modulus and phase measurements corresponding to the PDLC device can be represented in a Bode plot, where the trend of the impedance magnitude and phase can be analyzed. Figure 2 shows the experimental data of the magnitude and phase of impedance as a function of frequency, measured in the temperature range between 25 and 40 °C. Impedance results in all devices are similar and the small discrepancy should be due to differences in the preparation of the PDLC composites. Selecting the suitable frequency range for the operation of the devices is a matter of simplifying the PDLC response, and Figure 2 can contribute to making this choice. Low frequencies are discarded because a low frequency electric field can induce degradation of the LC material due to the adsorption of ion charges and generation of strong electric field on the electrode layers [9,10], and a strong dependence with the temperature is also observed in Figure 2 at those frequencies. Moreover, the suitable frequency range should be selected by choosing the range where the device shows a purely capacitive behavior, simplifying its response. In that range, there is a linear decay in magnitude and a phase close to −90 degrees [2].

Complex impedance measurements displayed in the Figure 2 reveal that there is a capacitive behavior displacement when increasing the temperature, but also that the working frequency range of 10^4^ to 10^5^ Hz should be the optimal one, since the temperature dependence is null both in the impedance value and in the electrical response, which is purely capacitive.

## 4. Electrical Equivalent Circuit Proposal

**Figure 3.**
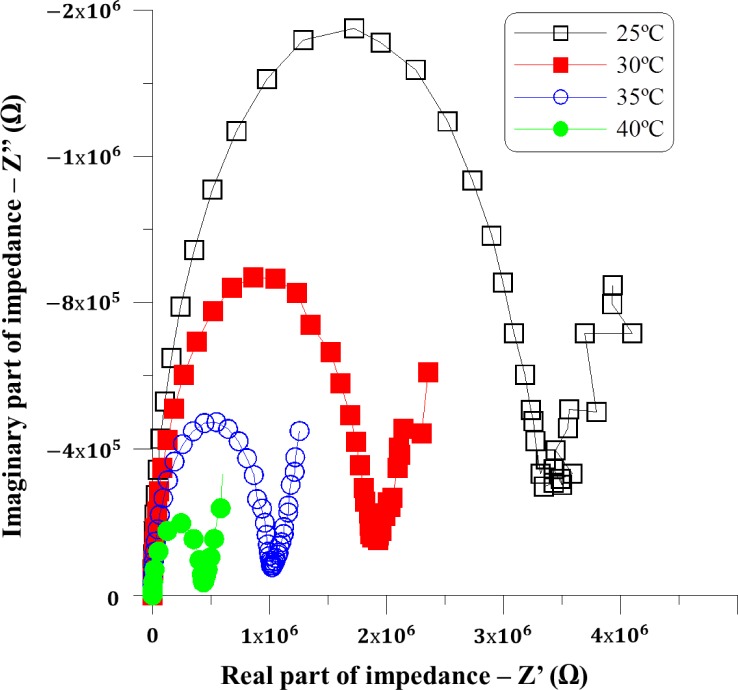
Impedance spectrum (Nyquist plot) for PDLC.

An equivalent electric circuit has been obtained in order to study the dependence of electrical components on frequency, voltage and temperature. Figure 3 shows a Nyquist plot of experimental impedance, where its real part is plotted on the X axis and its imaginary part on the Y axis, the shape of which shape suggests that there are two different regions with a distinct behavior [11].

The semicircle portion, due to impedance on frequencies from 10 to 10^7^ Hz, is usually related to the effect of charges stored in a capacitor which is discharging through a resistor. This discharge is produced with an exponential decay in time, being the diameter related to a time constant dependent on the resistor and capacitor values. Semicircle appearance means that there is only one time constant involved. This effect can be closely modeled by a Randles equivalent circuit.

**Figure 4.**
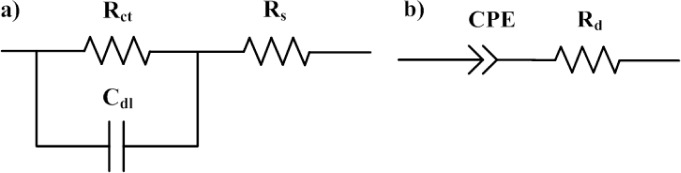
(**a**) Equivalent electric circuit for a frequency range from 10 to 10^7^ Hz; (**b**) equivalent electric circuit for a frequency range from 10^−1^ to 10 Hz.

The equivalent circuit for the Randles cell is shown in Figure 4a. This circuit includes a resistor *R_s_*, which represents the influence of electrodes, a double layer capacitor C_dl_ due to dipolar polarization and the symmetry of the device, which is composed of several layers, and a resistor *R_ct_* standing for the mobility of free charges and dipolar displacement inside the device.

At high frequencies, C_dl_ impedance is as low as it behaves as a short circuit, and all of the impedance is coming from *R_s_*. As it is a resistance due to electrodes, no apparent dependence with temperature is found at highest frequencies.

At low frequencies, C_dl_ impedance is as high as it behaves as an open circuit, and the main influence is *R_ct_*, which value is thus related with the diameter of the semicircle of Figure 3. As a resistance due to charge transfer or movement, it depends clearly on temperature.

Nyquist plots show also a straight tail with a certain slope which appears at low frequencies in the range of 10^−1^ Hz to 10 Hz. As it is shown in Figure 3, during this frequency range, the phase of the impedance, *i.e.*, the slope on the Nyquist plot, remains almost constant. These results are in good agreement with those showed in Figure 2, at low frequencies. Several researches have reported a similar behavior [9,10,12–14], where the constant phase is explained by the creation of a strong electric field on the alignment layers. Therefore, a constant phase element (CPE) has been included to model the phenomenon.

CPE is a simple distributed element which tends to behave as a capacitor and has a constant phase angle in the impedance. The electric equivalent circuit proposed for such situation is represented in Figure 4b. The model includes an association CPE in series with a resistance R_d_ due to dipolar displacement.

Based on the fact that each frequency range presents a different behavior, a combination of the two proposed different equivalent circuits must cover the whole range. The complex impedance Z for these equivalent circuits can be described by Equation (1) for higher frequencies and Equation (2) for lower ones:

$$Z{|}_{10<f<10^{7}}=R_{s}+\frac{R_{ct}}{1+s\cdot C_{\text{dl}}\cdot R_{ct}}$$ (1)

$$Z{|}_{10^{-1}<f<10}=R_{d}+\frac{1}{T\cdot s^{-P}}$$ (2)

**Figure 5.**
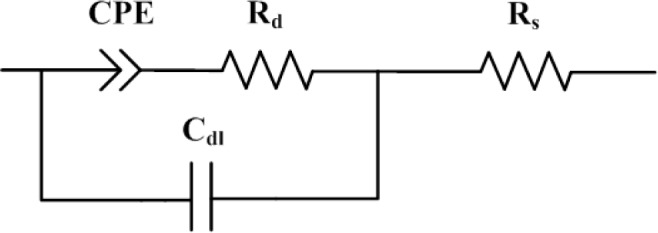
Equivalent electric circuit for a frequency range of 10^−1^ to 10^7^ Hz.

The coefficient T and the exponent P are the parameters of the CPE. Generally, 0 < P < 1; however, CPE tends to a response of a capacitor of capacitance T when P is equal to 1. As the frequency ranges could be separated in the electrical circuit effects, both electric circuits can be simplified in a single circuit when the low frequencies parameters (CPE and *R_d_*) are embedded in the *R_ct_* element. This proposal is shown in Figure 5, valid for the whole frequency range between 10^−1^ and 10^7^ Hz. The influence of each element will depend on the frequency range.

The following fit is done for the measurements at 25 °C. For high frequencies, the capacitive elements act as short circuit. Thus, the value of *R_s_* is equivalent to the impedance magnitude at highest frequency. This impedance has a value of 178 Ω (see Figure 2). On the other hand, the capacitance C_dl_ can be estimated using the impedance magnitude plot of Figure 1. At frequencies into the selected 10 to 100 kHz range, when the device has a purely capacitive behavior because the phase is close to −90°, C_dl_ should be the main contribution, and could be calculated using the following expression (derived from the one of a capacitor impedance):

$$C_{\text{dl}}=\frac{1}{2\cdot \pi \cdot f_{-90{}^{\circ}}\cdot |Z{|}_{-90}}$$ (3)

Therefore, the capacitances C_dl_ are 0.89 nF. Complex non-linear least squares (CNLS) was used to obtain the value of *R_d_* and the parameters of CPE, fitting the rest of the equivalent circuit parameters of Figure 5 with the impedance measurements, and using the Levenberg Marquardt nonlinear interpolation method [15]. The elements of the equivalent circuits are given in Table 1.

**Figure 6.**
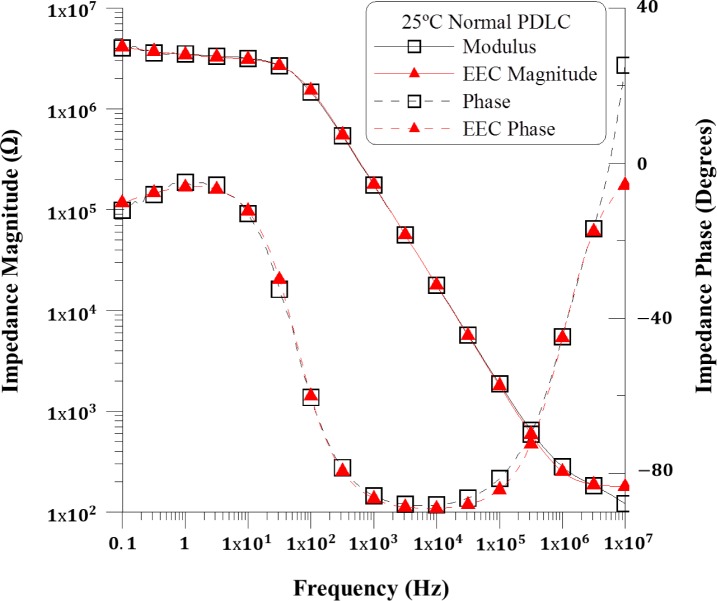
Experimental modulus and phase (squares-line) and simulated data (triangles-dashed line) for a frequency range from 10^−1^ Hz to 10^7^ Hz in the PDLCs devices.

The experimental magnitude and phase impedance were compared with the simulated data to validate the proposed equivalent electric circuit. This comparison is shown in Figure 6 as Bode plots. It can be inferred that the electric equivalent circuit proposed in Figure 5 satisfactorily reproduces the performance of impedance complex behavior for the PDLC devices under study in a wide frequency range. A slight discrepancy appears in the high frequency region, probably due to the inductance of the PDLC wires.

Once the equivalent circuit has been fitted with the impedance measurement for a given temperature, the evolution of the electrical response as the temperature increases has also been analyzed. The previous fitting is repeated for the measurements at every temperature. At high frequencies, the R_s_ element is the main influence in the electric circuit. At these frequencies the temperature dependence is null and does not lead to a change in the complex impedance; therefore, the evolution of the R_s_ is independent of temperature. Moreover, in order to analyze the influence with temperature, the C_dl_ parameter has been added in the fitting.

Table 2 shows the evolution of the retrieved fitted elements of the equivalent circuit with the temperature. These results are in good agreement with those reported previously in the diagram showing the experimental complex impedance as a function of frequency (Figure 2).

The influence of C_dl_ related to dipolar polarization is independent with temperature, whereas CPE-P and CPE-T parameters related to ion accumulation, increase with temperature. Therefore, as it is expected from the above discussion, PDLC has a less capacitive behavior when the temperature is increased. The exception is observed in *R_d_* parameter related to dipolar displacement.

On the other hand, the resistance *R_d_* value is now the one related to the diameter of the semicircle in the Nyquist plot. The Figure 3 shows that when temperature increases, the diameter of the semicircle decreases, and also the value of *R_d_* and thus the dipolar displacement. The C_dl_ element at the whole frequency range (10^−1^–10^7^ Hz) slightly changes with temperature, as the constant shape of the semicircle predicts. The straight tails of Nyquist plots at Figure 3 are related with the component CPE, and the slope depends on the parameter CPE-P. The slope increases with the temperature. This behavior can be reflected in the values of the CPE-P parameter in Table 2. Further characterizations must be done to explain this effect.

## 5. Conclusions

In this work, electrical properties of PDLCs have been studied as a function of temperature and frequency of the applied voltage. An electrical model is suggested and validated. Using the proposed model, many physical processes can be simulated, such as influence of electrodes, dipole movements contribution, ion charge accumulates and the effects of the temperature. Future research will focus on the simulation of optimized driving circuits and signals in order to reduce power consumption using this equivalent circuit.

## Figures and Tables

**Table 1.** Results of fitting presented in the Figure 5.

Parameter	Value	Error (%)
*R_d_* (MΩ)	3.01	1.7
CPE-T	9.06 × 10^−7^	4.7
CPE-P	0.37	8.8

**Table 2.** Results of fitting in the temperature range of 25 to 40 °C.

Parameter	Parameter@25 °C	Value	Error%
@25 °C	*R_d_* (MΩ)	3.00	1.72
	C_dl_ (nF)	0.89	0.45
	CPE-T	9.15 × 10^−7^	5.26
	CPE-P	0.37	8.89

@30 °C	*R_d_* (MΩ)	1.73	1.08
	C_dl_ (nF)	0.83	0.40
	CPE-T	1.51 × 10^−6^	3.82
	CPE-P	0.43	5.91

@35 °C	*R_d_* (MΩ)	0.96	0.71
	C_dl_ (nF)	0.82	0.40
	CPE-T	2.38 × 10^−6^	3.01
	CPE-P	0.55	3.69

@40 °C	*R_d_* (MΩ)	0.41	0.95
	C_dl_ (nF)	0.84	0.64
	CPE-T	3.39 × 10^−6^	3.07
	CPE-P	0.58	3.41
